# Effect of Growth Pressure on Epitaxial Graphene Grown on 4H-SiC Substrates by Using Ethene Chemical Vapor Deposition

**DOI:** 10.3390/ma8095263

**Published:** 2015-08-26

**Authors:** Shuxian Cai, Zhonghua Liu, Ni Zhong, Shengbei Liu, Xingfang Liu

**Affiliations:** 1 National Research Center of Engineering Technology for Utilization of Functional Ingredients from Botanicals, Hunan Agriculture University, Changsha 410128, China; E-Mails: caisx@bjmu.edu.cn (S.C.); liu.zhonghua2258@gmail.com (Z.L.); 546033652zn@gmail.com (N.Z.); 2 Key Laboratory of Semiconductor Materials Science, Institute of Semiconductors, Chinese Academy of Sciences, P.O. Box 912, Beijing 100083, China; E-Mail: liushengbei@semi.ac.cn

**Keywords:** face dependences, epitaxial graphene, chemical vapor deposition, 4H-SiC, growth pressure

## Abstract

The Si(0001) face and C(000-1) face dependences on growth pressure of epitaxial graphene (EG) grown on 4H-SiC substrates by ethene chemical vapor deposition (CVD) was studied using atomic force microscopy (AFM) and micro-Raman spectroscopy (μ-Raman). AFM revealed that EGs on Si-faced substrates had clear stepped morphologies due to surface step bunching. However, This EG formation did not occur on C-faced substrates. It was shown by μ-Raman that the properties of EG on both polar faces were different. EGs on Si-faced substrates were relatively thinner and more uniform than on C-faced substrates at low growth pressure. On the other hand, D band related defects always appeared in EGs on Si-faced substrates, but they did not appear in EG on C-faced substrate at an appropriate growth pressure. This was due to the μ-Raman covering the step edges when measurements were performed on Si-faced substrates. The results of this study are useful for optimized growth of EG on polar surfaces of SiC substrates.

## 1. Introduction

Epitaxial growth of graphene (EG) is more facile in area and layer thickness control [1] than the classic exfoliation of highly oriented pyrolytic graphite crystals (HOPG) [2]. Growth of wafer-scale and hundreds of square meters of mono-layer EG and few-layer EG has been demonstrated [3,4,5], which paves the way for modern semiconductor applications of graphene [6,7]. Shortly after HOPG was demonstrated by Novoselov K.S. and Geim A.K. [2], Berger C. [8] conducted EG on semi-insulting silicon carbide (SI SiC) by using sublimation of silicon atoms from SI SiC surface at high temperature (S-EG). This method has been proved to be efficient for wafer-scale EG-based microelectronics [9,10]. However, S-EG based on SiC wafer is high energy-consumption and expensive since the thermal decomposition is performed at a temperature higher than 1600 °C using the expensive commercial available SiC wafer as the starting material. An alternative solution is growth of graphene on metal foils, which sharply reduces the process cost because the growth process is performed at a temperature about 1000 °C using metal foil as the starting material. The metal foil, especially industrial Cu foil, is cheap even for hundreds of square meters, which is suitable for large-scale EG fabrication [11,12]. On the other hand, polycrystalline metal foils are conductors, which leads to a necessary transfer process of EG from metal foil to target substrate such as SiO_2_/Si [13]. The transfer process is a key issue for EG and has been intensively studied. EG transferred from metal foils are polycrystalline, with grain boundaries being random distribution, which hampers the electrical transport properties [14]. In contrast, SiC wafers are semiconductors or semi-insulting, which means that it is not necessary for EG on SiC to be transferred to other target substrates. In addition, SiC substrates are monocrystalline; nano-sized steps appear in the surface of SiC after high temperature annealing [15]. The steps are like ladders, which guide graphene grown on SiC surface smoothly and flatly [16]. Therefore, the electrical transport properties of EG on SiC are superior to those of EG from metal foil [17].

Recently, EG growth on SiC by using chemical vapor deposition (CVD) has been developed [18]. In this growth process, graphene is synthesized by thermal decomposition of carbon precursor instead of sublimation of surface Si atoms from SiC substrate. By both using carbon precursor and controlling the dynamic flow in the chamber [11], various reaction parameters in affecting the number of layers and the defect degree of the synthesized graphene have been reported, such as annealing time, heating ramp rates [19], hydrogen partial pressure [20], and growth time [21]. Among them, EG by CVD process is more versatile than EG by sublimation of Si atoms from SiC surface. For example, CVD process enables the growth rate on SiC substrate lower enough to growth of 1 ML EG [18], which is extremely difficult to achieve in the case of S-EG. Additionally, the CVD process enables doped EG grown on the SiC substrate, which creates more opportunities for the study of graphene applications.

Generally, the surfaces of commercial available nominally on-axis 4H-SiC substrates are Si-terminated or C-terminated. This means that 4H-SiC has two polar faces, *i.e.*, C-face and Si-face. It has been reported that graphene can be grown on Si- and C-faced substrates, respectively [22]. In this paper, we report the CVD growth of EG on both Si- and C-faced 4H-SiC substrates using ethene gas as the carbon precursor. Our proposed approach enables simultaneous growth of EG on Si- and C-faced SiC by both adjusting the flow rate of the carbon precursor and the chamber pressure. In this way, we are able to investigate the growth nature of CVD EG on both polar faces of 4H-SiC.

## 2. Results and Discussion

**Figure 1:**
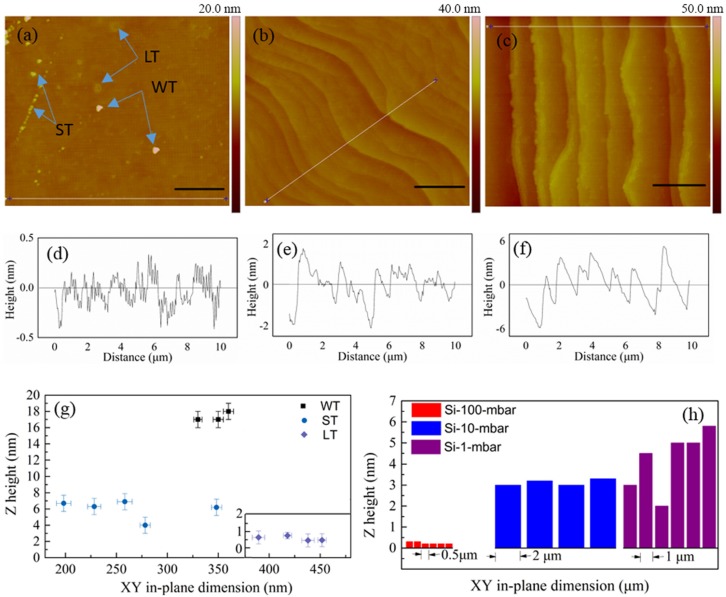
Epitaxial graphene (EG) samples grown on Si-faced 4H-SiC substrates. (**a**) Sample Si-100-mbar; (**b**) Sample Si-10-mbar; (**c**) Sample Si-1-mbar; (**d**–**f**) Atomic force microscopy (AFM) line profiles for the white lines in (**a**), (**b**) and (**c**), respectively; (**g**) Distribution of three dimensional sizes of individual terraces in (**a**); (**h**) Histograms of steps and terraces sizes of EG surfaces on Si-faced substrates. Scale bars: 2 μm.

Figure 1 shows the AFM morphologies of EG on Si-face 4H-SiC substrates. The growth pressure plays an important role on the EG morphologies. When the pressure is set to be 100 mbar, which is the highest pressure in our experiments, on the whole the EG surface is smooth, without obvious features except for some individual terraces (Figure 1a). However, small steps of ~0.4 nm height still can be observed (Figure 1d). Terraces in Figure 1a can be categorized into three types: white triangle ones (WT), small triangle ones (ST) and light ones (LT). The arrows in Figure 1a point to these terraces. The sizes of these terraces are distinct. WT is the thickest one while LT is the thinnest one; ST is the smallest one, but LT is the largest one (Figure 1g). The thickness of LT is 0.6~0.8 nm, which is about the thickness of 2 ML graphene [23].

When the growth pressure decreases, the surface morphologies of the EG are mainly dominated by large steps (Figure 1b,c). These steps are regular, and the mesas between them are covered with small steps. The height of large step increases with growth pressure decrease (Figure 1h), but the height of the small step remains almost in the same range, with a typical value of ~0.5 nm (Figure 1e,f). The widths of the mesas between the steps also vary according to growth pressure. The mesa width of sample Si-10-mbar is ~2 μm, which is about twice of that of Si-1-mbar and four times of that of Si-100-mbar (Figure 1h). The presence of large steps is mainly due to growth at relatively low pressure and high flow rate [18].

**Figure 2:**
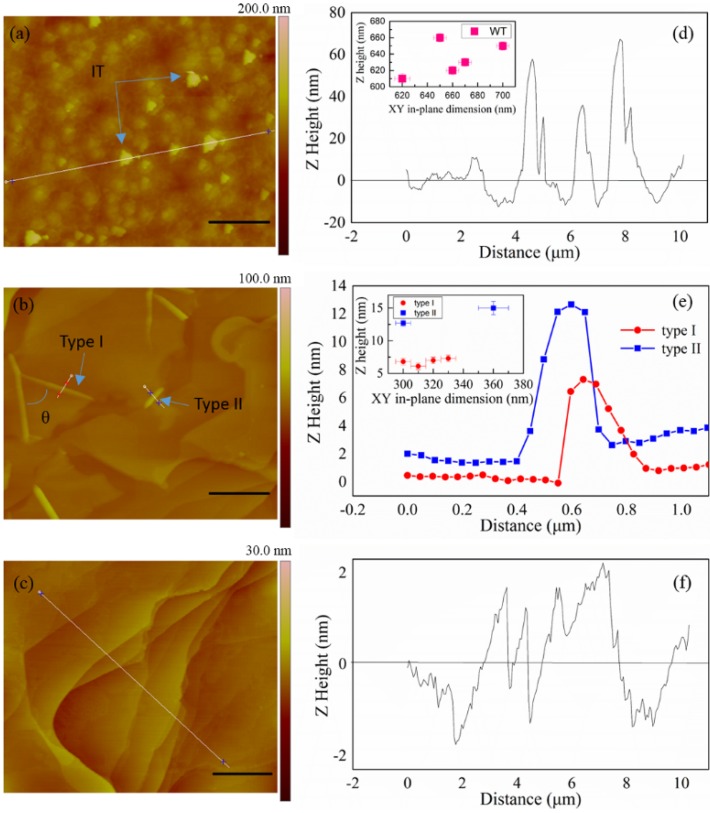
EG samples grown on C-faced 4H-SiC substrates. (**a**) Sample C-100-mbar; (**b**) Sample C-10-mbar; (**c**) Sample C-1-mbar; (**d**) Atomic force microscopy (AFM) line profile for the white line in (**a**). Inset: distribution of three dimensional sizes of individual terraces in (**a**); (**e**) AFM line profiles for two types of ridges in (**b**). Inset: distribution of three dimensional sizes of ridges in (**b**); (**f**) AFM line profile for the white line in (**c**). Scale bars: 2 μm.

The growth results are distinct at C-faced 4H-SiC substrates. When the growth pressure is set to be 100 mbar, the surface becomes rough (Figure 2a), with an uneven distribution of irregular terraces (IT). The in-plane sizes of the ITs are almost double of that of the WTs (Figure 1g and Figure 2d), and the height of IT is 50~70 nm, which is obviously larger than that of WT. These results show that the efficiency of carbon deposition on C-faced substrate is higher than on the Si-faced substrate at the same growth conditions.

When the growth pressure decreases to 10 mbar, the surface morphologies of the substrate are mainly overlapped by large EG layers. The EG overlayers form flat facets that continuously coat the SiC substrate. There are ridges in some EG. The ridges can be categorized into type-I and type-II ridges. The arrows in Figure 2b point to these ridges. The type-I ridges originate from ridge nodes, as reported by other literatures [24]. A type-I ridge and a type-II ridge is distinction as follows: (i) the maximum height of a type-I ridge is typically less than the height of a type-II ridge (Figure 2e); (ii) a type-II ridge often appears alone, like a short straight line, while type-I ridges often appear as triplets, with a certain intersection angle, and form a crease-like distortion on the surface of a flat graphene facet. The type-I ridges are interconnected, often diverging from a well-defined ridge node by forming a subtended angle *θ*. In Figure 2b, *θ* shows preference values of 60° and 120°. These angles are close to high-symmetry directions in the graphene lattice, therefore, it can be deduced that ridge tends to occur along high-symmetry directions in the EG films [24]. When the growth pressure further decreases to 1 mbar, the surface morphologies of the EG are mainly dominated by steps, but the steps are unobvious and irregular (Figure 2c), with a height of 2.5~3 nm (Figure 2f), which indicating EG on C-faced substrate is more complicated than on Si-faced substrate.

To characterize the EG on both the Si- and the C-faced substrates, we perform Raman measurements. Micro-Raman spectroscopy is a non-invasive but powerful technique for graphene characterization [25]. Raman bands at ~1580 cm^−1^ (G band) and ~2700 cm^−1^ (2D band) are characteristic peaks for defect-free graphene; band at ~1350 cm^−1^ (D band) is characteristic for graphene defects such as grain boundaries and edges. The G band originates from in-plane vibration of 𝑠𝑝^2^ carbon atoms [26] and the 2D band originates from the double-resonance processes of the two phonons with opposite momentum in the highest optical branch near the 𝐾 points in the Brillouin zone [27]. Because the second-order Raman bands of 4H-SiC sit in the range of 1400~1800 cm^−1^ [28], the D and the G band of graphene on 4H-SiC are complicated. It is necessary to subtract a pristine 4H-SiC Raman spectrum from spectra of EG on 4H-SiC substrates to reveal the graphene spectra [29].

**Figure 3:**
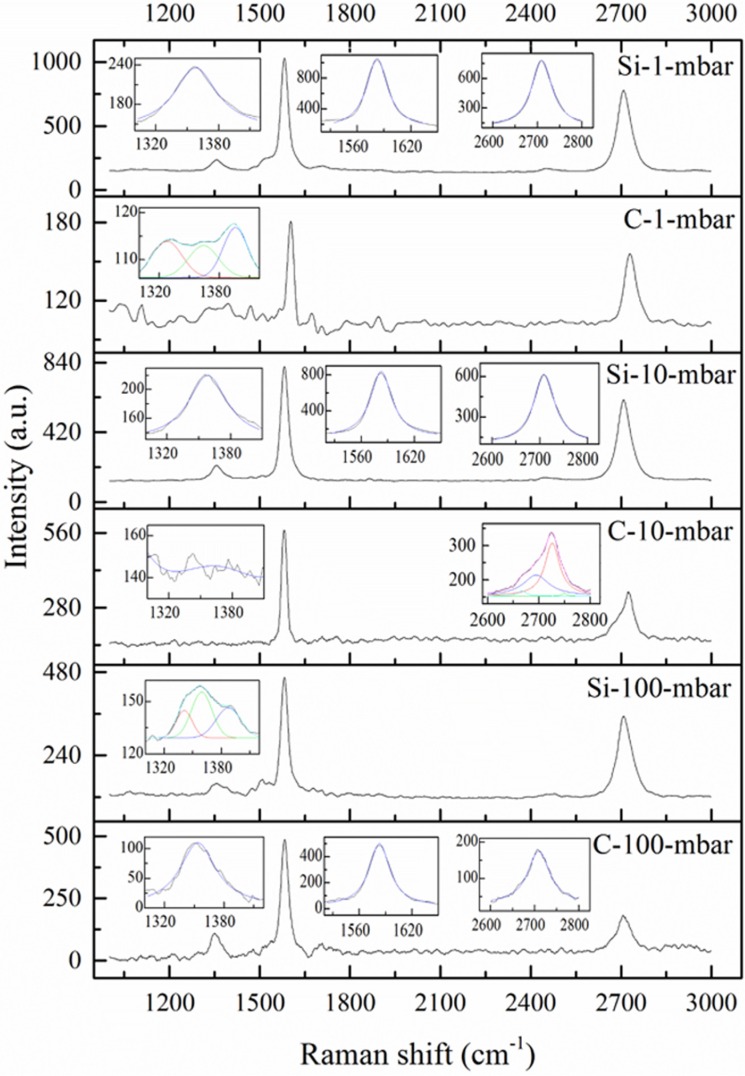
μ-Raman spectra of EG on Si- and C-faced 4H-SiC substrates. The D, G, and 2D band peaks are marked. Insets: Lorentz fits to characterize the width and position of Raman peaks.

Figure 3 shows typical Raman spectra of six EG samples. The Lorentz fitted D, G and 2D peaks are also shown in Figure 3 as insets. The frequency integrated intensity ratio of the D to G peak (denoted as A_D_/A_G_) and the corresponding intensity ratio (I_D_/I_G_) are used as characteristic parameters for graphene characterization. There is difference between A_D_/A_G_ and I_D_/I_G_ in certain situation [30]. The former is suitable for characterization of graphene with small disorder or perturbations. This is due to the fact that the integrated intensity of each peak represents the probability of the whole Raman process, which considering uncertainty [31]. The latter represents the phonon modes/molecular vibrations involved in the Raman process [32] and is suitable for characterization of graphene with large disorder. However, it is better for I_D_/I_G_ to combine with full width at half-maximum (FWHM) of Raman peaks when analysis performed since FWHM is a measure of structural disorder [33].

D peaks of sample C-100-mbar, Si-10-mbar and Si-1-mbar are symmetrical and can be fitted by one Lorentz peak, respectively. However, D peaks of sample C-1-mbar and Si-100-mbar are asymmetrical; they can be fitted by three Lorentz peaks, respectively. The D peak of sample C-10-mbar does not appear, which indicating the high quality of EG on C-faced substrate at growth pressure of 10 mbar. The G peaks of all samples can be well fitted by one Lorentz peak, so it is easy to calculate the A_D_/A_G_ and the I_D_/I_G_ for sample C-100-mbar, Si-10-mbar and Si-1-mbar, respectively. However, it is out of the question to calculate these values for sample C-1-mbar and Si-100-mbar since they are fitted by multiple Lorentz peaks. The fitting values and the calculated results are listed in Table 1. The values of A_D_/A_G_ are larger than that of I_D_/I_G_, by a factor of 1.3~1.4. However, the change direction of both A_D_/A_G_ and I_D_/I_G_ is the same: increasing with increased growth pressure. Judging by the I_D_/I_G_ ratios of Si-1-mbar, Si-10-mbar and C-100-mbar, high growth pressure can result in the decrease of EG quality. However, this cannot be explicitly applied to C-1-mbar, C-10-mbar and Si-100-mbar, for no I_D_/I_G_ ratios of these samples are available.

G peaks of all samples are almost located at the same position, 1582 ± 1 cm^−1^, except that of C-1-mbar, 1602 ± 1 cm^−1^ (Table 1). D peaks of Si-1-mbar and Si-10-mbar are located at 1358 cm^−1^. D peak of Si-100-mbar is a multi-peak, which can be fitted by three Lorentz peaks, and the center Lorentz peak is located at 1359 cm^−1^, almost as the same value as that of Si-10-mbar. This means that no Raman shift is seen for graphene grown on Si-faced substrates. Graphene grown on C-faced substrates is more complicated. Although G peaks of C-1-mbar, C-10-mbar and C-100-mbar are not at the same position, they do not take a steady change in direction which is related to the growth pressure.

**Table 1:** Results of Lorentz fits and grain sizes calculation for EG on polar faces of 4H-SiC substrates.

Samples	D (cm^−1^)		G (cm^−1^)		Ratio		La (nm)	
	X	FWHM	X	FWHM	I_D_/I_G_	A_D_/A_G_	(I_D_/I_G_)	(A_D_/A_G_)
Si-1-mbar	1358	50	1582	29	0.10	0.18	167.5	93.1
C-1-mbar	/	/	1602	30	----	----	----	----
	1328	35	/	/	----	----	----	----
	1364	35	/	/	----	----	----	----
	1396	29	/	/	----	----	----	----
Si-10-mbar	1358	45	1583	30	0.12	0.19	139.6	88.2
C-10-mbar	/	/	1581	21	----	----	----	----
Si-100-mbar	/	/	1582	28	----	----	----	----
	1341	20	/	/	----	----	----	----
	1359	25	/	/	----	----	----	----
	1387	30	/	/	----	----	----	----
C-100-mbar	1354	44	1583	29	0.22	0.33	76.1	50.8

Notes: /: unavailable; ----: no result; X: peak position; (I_D_/I_G_): La is calculated by taking I_D_/I_G_ value.

The average grain sizes La can be evaluated according to the empirical relation: La = (2.4 × 10^−10^)·λ^4^(I_D_/I_G_)^−1^ [30] or La = (2.4 × 10^−10^)·λ^4^(A_D_/A_G_)^−1^ [34], where λ is the wavelength of the excitation laser. The value of La contains information about the average scattering distance; it represents the average distance of the randomly distributed defects in disordered graphene [34]. It is estimated that the La of sample C-100-mbar, Si-10-mbar and Si-1-mbar is 76.1 nm, 139.6 nm and 167.5 nm, respectively. The La value of 167.5 nm is closed to literature reported value [35], which indicates the high quality of Si-1-mbar.

## 3. Experimental Section

Graphene was grown on the Si- and the C-face of on-axis 4H-SiC substrates, respectively, using a custom-made vertical CVD (Mass Electronic Inc., Hefei, China) hot-wall reactor inductively heated by an RF generator (Mass Electronic Inc., Hefei, China) [15]. The substrates were 10 × 10 mm^2^ pieces diced from a single wafer with both faces epi-ready polished. For each run, two substrates (one Si- and one C-face substrate) were placed onto a SiC-coated high purity graphite suscepter side by side and then loaded into the growth chamber. The suscepter was rotated by a motor with a rate of 50 rpm. This made the difference of growth factor for the two substrates minimum. The substrates were hydrogen etched at 1500 °C for 30 min to remove oxides and polishing scratches before growth. After that, the chamber was pumped until the pressure was down to ~1 mbar, and the substrate temperature was ramped to 1600 °C in 5 min for graphene growth. The temperature was measured by a pyrometer facing directly to the substrate. In this study, C_2_H_4_ diluted in H_2_ was used as feed gas; Ar was used as carrier gas. The chamber pressures were set to be 1, 10 and 100 mbar, respectively, for three individual runs, and the corresponding samples are denoted as Si-1-mbar, Si-10-mbar, Si-100-mbar and C-1-mbar, C-10-mbar, C-100-mbar, respectively, for EG on Si-faced substrates and C-faced substrates. After a growth duration of 10 min, C_2_H_4_ and Ar were turned off. The RF generator was shut off and the chamber cooled naturally to room temperature under H_2_ ambient.

All samples were characterized by atomic force microscopy (AFM) (Veeco Instruments, NewYork, NY, USA) at room temperature. Graphene was confirmed by Raman spectroscopy (model LabRAM HR Evolution, Horiba Jobin Yvon, Paris, France). Micro-Raman scattering measurements were performed in a backscattering geometry at room temperature under the Ar ion laser at a wavelength of 514.5 nm (2.41 eV). The laser spot diameter on the sample surface was approximately 1 μm. To avoid surface heating, the excitation power of the laser was limited to be 10 mW.

## 4. Conclusions

In summary, we have studied the epitaxial graphene (EG) grown on polar surfaces of 4H-SiC substrates at different growth pressure by ethene chemical vapor deposition using AFM and μ-Raman measurements. The results show that the morphology, quality and thickness of EG on Si- and C-faced substrates are quite different due to their distinct growth nature. This study is helpful for the growth of EG on polar surfaces of 4H-SiC substrates with improved structural quality.
